# The relationship between family resilience and the psychological well-being and life satisfaction of pregnant women: the mediating role of individual resilience

**DOI:** 10.1186/s40359-024-01547-6

**Published:** 2024-02-06

**Authors:** Majid Yousefi Afrashteh, Parinaz Hanifeh, Zekrollah Morovati

**Affiliations:** Department of Psychology, Faculty of Humanities, University of Zanjan, Zanjan, Iran

**Keywords:** Family resilience, Life satisfaction, Pregnant women, Individual resilience, Psychological well-being

## Abstract

**Background:**

Pregnancy period is an important experience in the life process of married women, which leads them to growth and development and is considered as a part of the puberty process. The aim of this research is to determine the mediating role of individual resilience in relation to family resilience, psychological well-being and life satisfaction of the pregnant women. The current study is correlational according to the practical purpose and based on data collection.

**Methodes:**

The study population of the current research is all pregnant women in 2021, and 361 of them responded to the self-report questionnaires of family resilience, psychological well-being, life satisfaction, and individual resilience. To analysis the model, the path analysis method was used utilization spss-26 and Lisrel 10.2 software.

**Results:**

The results show a good fit of the model with the data. The results also showed a direct and significant effect between life satisfaction with obligation (β = 0.22 and t = 3.42), with challenge (β = 0.28 and t = 3.98), with control (β = 0.11 and t = 2.13), psychological well-being with obligation (β = 0.20 and t = 3.11), with challenge (β = 0.20 and t = 2.73) and with control (β = 0.45 and t = 10.34).

**Conclusion:**

The upshot of this research can be considered in interventions related to pregnant women. Strengthening resilience in this group can be useful for increasing life satisfaction and psychological well-being of pregnant women.

## Background

Pregnancy is considered a natural process in the life of married women [1], which is an important stage of development and growth, where about two-thirds of married women get pregnant at least once in their married life [2]. Pregnancy is a period with lots of pleasure and stress [3] And due to psychological, physiological and hormonal changes, women have become more affected and sensitive to negative life events [4] that being exposed to the risk and the wrong way of tolerance and compromise with these adversities can produce serious problems in the health of women and fetuses [5] During pregnancy, due to numerous changes, women have an urgent need for family support, so that they can solve their problems and reduce stress [3].

According to the definition of the World Health Organization, mental health or psychological well-being it refers to a state in which a person realizes his abilities and copes with the pressure and problems of life and can contribute to his society [6] Some researchers define psychological well-being as pleasurable experiences, feelings of happiness, vitality, and the absence of life pressures [7, 8] that people’s personal experiences and achieving goals can include this feeling; In fact, it refers to the degree of control over the meaning and concept of life and activities related to it [7] Emotional and cognitive support from the family is considered as an important factor to strengthen them [9].

These days, many essential factors predict people’s mental health, of which life satisfaction is a part of it. Life satisfaction is a part of mental well-being [10] and a unique component for people. It is kind of cognitive evaluations of personality and different aspects of life are included and are based on the principle of personal judgment [11, 12], and the lack of balance at work, multiple duties and responsibilities in life can reduce this feeling [13]. It shows the balance and difference between the objective and ideal situation, including the person’s satisfaction in the past, present and possible future, and the stage of desire to continue the life [14].

Compromising with environmental changes and events and creating a sense of balance is one of the important abilities of humans, which is done with the help of a structure called resilience [15, 16], and it is mentioned as protective and vital factors against adversity and crises in life, which can reduce the effects of challenges [17, 18]. The element of resilience is a multidimensional component that is created under the influence of individual characteristics and environmental factors [16] and is defined as the capacity to adapt after a critical situation and a unique ability that varies from person to person [19]. Resilience is a dynamic and adaptive structure that creates adaptation and improvement of mental health conditions in critical situations and inappropriate situations [20, 21]. As the level of resilience increases in people, it can maintain physical and mental health by neutralizing the negative consequences of crises and, on the other hand, improve psychological well-being [22].

In addition to individual resilience, the concept of family resilience is also important because it is responsible for the group and support of members in dealing with life’s challenges and crises [23] And it is a belief system and a communication process between family members that explains it when faced with critical situations [24] and is considered as a positive strength for the family [11, 25] It can be said that this ability did not work at the moment, but it strengthens the abilities in the long term to resist and overcome future issues [26] And when the family successfully adapts to the crisis situation, this success can improve the confidence and trust of the family [27].

A review of the research history shows that pregnancy is one of the most important and evolutionary stages in the life of parents, which can create well-being in couples [28, 29]. On the other hand, the transition to motherhood is one of the most dangerous changes for women, the disturbances and problems in life can have negative effects on the mental health of the mother, fetus and baby, after birth, which has the ability to endure as a buffer and factor. It is a protection against negative effects that works directly and indirectly [3]. Aivalioti and Pezirkianidis [30] in their study, showed that parents who adopt appropriate strategies to deal with problems have a higher level of resilience and psychological well-being. The results of the study by Jain and collogues [4] showed that the family atmosphere and the intimacy with the spouse and the affection between the couple have a significant effect on their resilience and also their well-being, and it causes positive feelings in pregnant women, on the other hand, the occurrence of disagreements during pregnancy. It has a direct effect on discomfort and depression, which can reduce the feeling of life satisfaction in women’s world. Leon and collogues [5] conducted a study, the results of which showed that pregnant women with high resilience have lower levels of stress and psychological symptoms and higher levels of mental health, so finally it can be said that resilience and well-being They have become an important part of the factors related to the quality of health, and as the level of resilience improves, people feel more satisfied with life [28].

**Fig. 1:**
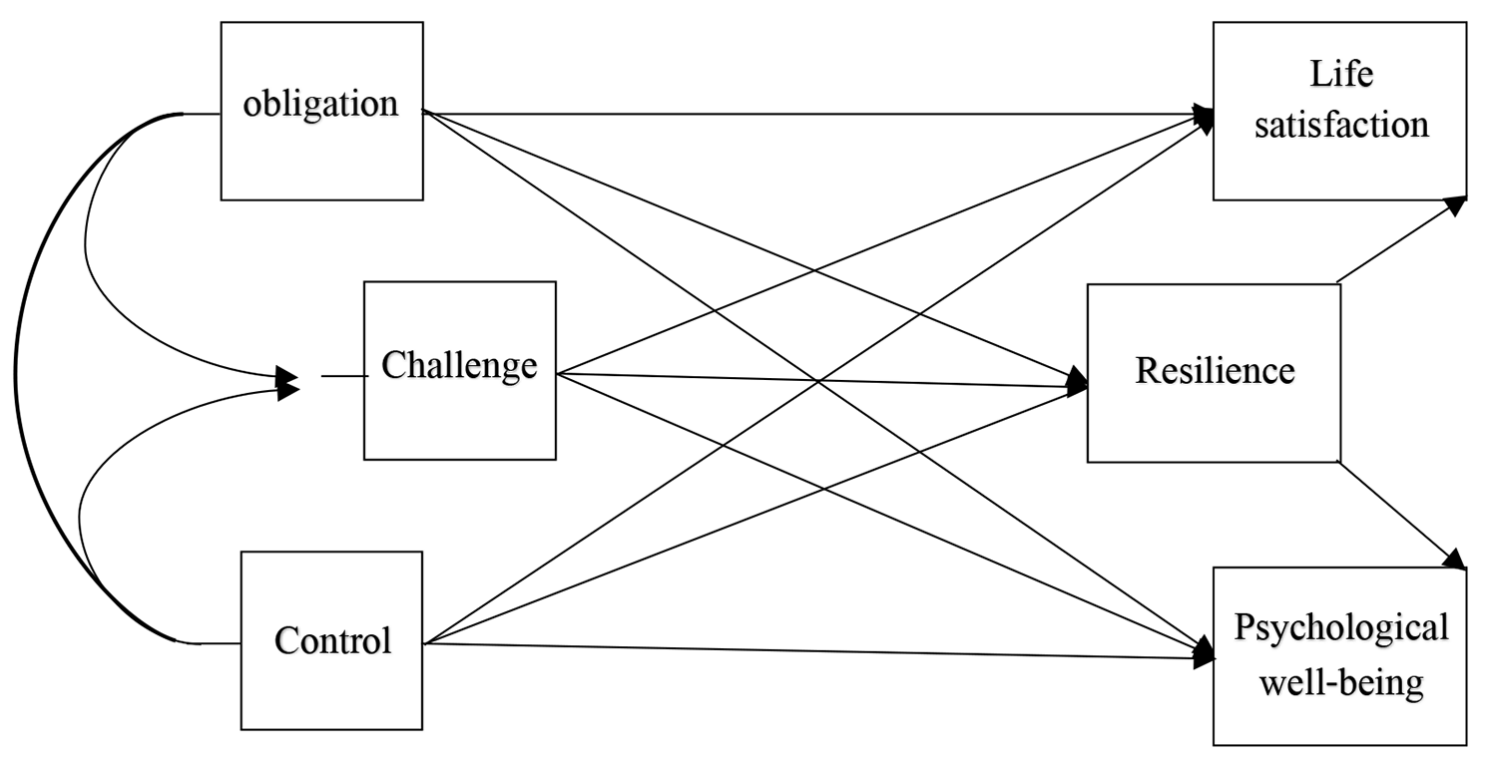
Conceptual model of the research

During the studies conducted on the current variables, studies have been conducted on the sample of students, the elderly and adults, but few studies have been conducted on pregnant women, because this group of people is a vulnerable group in our society due to sensitivity. are considered acceptable. Hormonal, physical and psychological changes are known. As mentioned, it is necessary to pay attention to research, and this category of women needs attention and mental and physical care more than other people. The more satisfied pregnant women are in the family, they can be a factor in the proper development of the fetus and ensure the health of their child in the future because any defect during pregnancy will have adverse effects on the future of the child. Therefore, it is necessary to conduct a research to identify the important and influential factors in the mental health of pregnant women and their life satisfaction, so the purpose of this research is family resilience with psychological well-being and life satisfaction with mediation analysis. The role of individual resilience in pregnant women, the theoretical model is shown in Fig. 1.

## Research hypotheses

1. Family resilience has a direct relationship with individual resilience in pregnant women.
2. Family resilience has a direct relationship with the psychological well-being of pregnant women.
3. Family resilience has a direct relationship with life satisfaction in pregnant women.
4. There is a direct relationship between individual resilience and psychological well-being in pregnant women.
5. There is a positive and significant relationship between individual resilience and life satisfaction in pregnant women.
6. Dimensions of psychological well-being significantly and positively predict life satisfaction in pregnant women.
7. Individual resilience plays a mediating role in the relationship between family resilience and psychological well-being in pregnant women.
8. Individual resilience plays a mediating role in the relationship between family resilience and life satisfaction in pregnant women.

## Methods

### Participant and data collection

The present cross sectional study was conducted on pregnant women referring to health centers, hospitals and private women’s medical offices in the third quarter of 2022 in Zanjan, Iran. To conduct this research, after obtaining the approval of the Department of Psychology and the approval of the National System of Ethics in Biomedical Research with code IR.ZNU.REC.1400.005 and obtaining the necessary permits, we referred to the comprehensive health service centers of Zanjan, Iran. The sample size was determined according to the Cochran’s formula for a population with an unknown number of 361 people. Pregnant women had been participated in this study from October 12, 2021 to January 10, 2022. To collect data, 8 out of 20 health service centers and 12 out of 25 private women’s medical offices in zanjan, iran were randomly selected. The centers were selected in such a way that the diversity of the socio-economic class was respected. After determining the sampling location, the interviewers came to these centers and asked the clients who met the criteria to enter the research to participate in completing the research questionnaires. Before completing the questionnaires, the participants were informed about the objectives of the research and their written informed consent was obtained. The inclusion criteria in this study were the age of the participating women, who were between 16 and 48 years old, pregnant women who decided to keep the pregnancy, no multiple pregnancy and no history of medical, obstetrical complications, and written consent. exclusion criteria in this study was more than 20% non-responced to each of the questionnaires.

#### Informed consent

was obtained from all participants and their legal guardians. Considering the possibility of non-return of some questionnaires, 400 women who were eligible to enter the study were asked to participate in the study. After data screening, the data of 39 participants were discarded due to outliers and non-responce, and finally the data obtained from 361 woman were analyzed. According to adequate power for Structural Equation Modeling (SEM), this was more than the suggested sample size [31] and this indicates that sample size provided adequate power. Informed consent was obtained from all participants and their legal guardians in accordance with the Declaration of Helsinki. All methods were performed in accordance with the relevant guidelines and regulations. Demographic information, including age, education, job, pregnancy weeks, type of pregnancy and number of pregnancy are presented in Table 1.

**Table 1:** Demographic Characteristics of the Studied Subjects

Variable	Number	Percentage
**age**		
16–20	23	6.37
21–25	78	21.61
26–30	97	26.87
31–35	119	32.96
36–40	32	8.86
41–45	11	3.05
46–50	1	0.28
**education**		
High school	81	22.44
Diploma	128	35.46
Associate degree	20	5.54
Bachelor’s	102	28.25
Masters	24	6.65
P.H.D	6	1.66
**Job**		
Employed	54	14.96
Housewife	307	85.04
**Pregnancy weeks**		
1–10	55	15.24
11–21	97	26.87
22–32	119	32.96
33–42	90	24.93
**Type of pregnancy**		
Intended	301	83.38
Unintended	60	16.62
**Number of pregnancy**		
First	157	43.49
Second	121	33.52
Third	72	19.94
Fourth	8	2.22
Fifth	3	0.83

### Measurements

#### Family Resilience Questionnaire (FHI)

The family resilience index was created in 1986 by McCubbin and his collogues [40] in order to evaluate the families resilience in dealing with stress. This test has 20 statements and has 3 subscales of obligation, challenge and control. The scoring method of this test is four-point Likert scale false (0), almost false [1], almost true [2], completely true [3], which is done in reverse in some expressions, but to obtain a sufficient overall score from the points related to all Add 20 phrases together. In this tool, the lowest score is 0 and the highest score is 60 The family resilience index has good reliability and its alpha coefficient is reported as 0.82. In the study of Persson, Benzein and Arestedt [32], the reliability coefficient was obtained by Cronbach’s alpha method for the total score of the tool at 0.86. Cronbach’s alpha in the research was obtained for obligation subscale 0.77, challenge subscale 0.79 and control subscale 0.81 and for the overall scale 0.84.

#### Psychological well-being questionnaire

The 18- questions short version of the psychological well-being scale designed by Ryff [33] and revised in 2002. This questionnaire has 18 questions and its purpose is to evaluate and examine psychological well-being from different dimensions of independence, mastery over the environment, and personal growth. Positive communication with others is purposefulness in life and self-acceptance. The scoring method is based on the 6-point Likert scale, which ranges from 1 to 6 from completely disagree to completely agree. The lowest value is 18 and the highest value is 108 in this tool. Cronbach’s alpha coefficient in Lee, Sun and Chiang’s research [34] was 0.88 and Cronbach’s alpha value was 0.86 in the current research.

#### Life satisfaction scale (SWLS)

This scale was created by Diener and his collogues [35] to measure the components of life satisfaction. That scale is a 5-item tool designed to measure a person’s cognitive and general judgment of life. The life satisfaction scale (SWLS) is scored on a 7-point Likert scale (1 totally disagree to 7 totally agree). The overall score of this scale is calculated by summing the answers and its range is between 5 and 35. A higher score indicates higher levels of life satisfaction. In the study of Merino, Privado and Duran [36], Cronbach’s alpha value for this scale was 0.88. In this study, Cronbach’s alpha value was 0.83.

#### Individual Resilience Questionnaire

The long form of the resilience scale was introduced by Wagneild [37] to measure individual resilience. The original form of the 25-item resilience scale was designed by Wagneild and Young in 1993 [38]. The short form of the individual resilience scale includes 14 questions out of the 25 questions in the long form. The scoring of this questionnaire is obtained directly according to the five-point Likert spectrum and with the total scores from completely disagree [1] to completely agree [5], Different researchers have used the short form scale and reported good validity and reliability on it. The minimum and maximum score in this tool is in the range of 14 to 70. Cronbach’s alpha was used to check the reliability of the scales, and in Shi et al.‘s research, Cronbach’s alpha was 0.94 [39]. In this study, Cronbach’s alpha was 0.75.

### Statistical analysis

Statistical analyzes for this study were performed using IBM SPSS 26 statistical software (IBM SPSS Statistics for Windows, 2019) on 361 participants with complete data. Descriptive statistics were calculated for demographic and research variables. Pearson’s correlation coefficient was used to measure the relationship between variables. Pearson’s correlation was used to test the relationship between three variables of family resilience, psychological well-being(PWB) and life satisfaction(LS). Then, a path analysis was conducted to examine whether individual resilience is a mediator between life satisfaction, psychological well-being and family resilience. Path analysis was performed using LISREL-10.2 with maximum likelihood estimation. *P* < 0.05 significance criterion was used for all statistical analyses.

## Results

Table 2 shows descriptive information including mean and standard deviation for research variables. In addition, Pearson correlation is reported to determine the relationship of all variables in the path model. For example the mean and standard deviation of obligation from family resilience variable are 9.36 and 18.68, respectively. The correlation coefficient of obligation subscale with indivitual resilience is -0.02, with life satisfaction is 0.30 and with psychological well-being is 0.45. More details are shown in Table 2.

**Table 2:** Correlation Matrix Statistics related to the Investigated Variables

Variable		Mean	SD	1	2	3	4	5	6
Family resilience	obligation	9.36	18.68	1					
	Challenge	4.98	16.86	0.73^**^	1				
	Control	6.32	21.76	0.34^**^	0.37^**^	1			
Individual resilience		16.90	57.33	0.02-	0.05	0.06	1		
Life satisfaction		6.69	25.49	0.30^**^	0.33^**^	0.24^**^	0.29^**^	1	
Psychological well-being		14.80	66.35	0.45^**^	0.46^**^	0.58^**^	0.13^*^	0.28^**^	1

** Correlation is significant at the 0.01 level. (two range test)

* Correlation is significant at the 0.05 level. (two range test)

The results of path analysis using Lisrel 10.2 software to determine the relationships between variables and investigate the direct and indirect effects are reported in Table 3.

**Table 3:** Direct, Indirect and Total Effects for the Relationship of the Variables

Path	Standard estimate		t-value	*p*-value
**Direct effect**				
Obligation→Resilience	0.183		2.896	*P* < 0.01
Challenge→Resilience	0.245		3.119	*P* < 0.01
Control→Resilience	0.056		1.107	*P* > 0.05
obligation→LS	0.170		2.652	*P* < 0.01
Challenge→LS	0.218		2.900	*P* < 0.01
Control→LS	0.110		2.138	*P* < 0.05
Obligation→PWB	0.173		2.856	*P* < 0.01
Challenge→PWB	0.173		2.826	*P* < 0.01
Control→PWB	0.453		10.343	*P* < 0.001
Resilience→LS	0.289		5.898	*P* < 0.001
Resilience→PWB	0.151		3.091	*P* < 0.01
**Indirect effect**				
Obligation→Resilience→LS	0.052		2.533	*P* < 0.05
Challenge→Resilience→LS	0.070		3.730	*P* < 0.05
Obligation→Resilience→PWB	0.037		1.993	*P* < 0.05
Resilience→PWB→Challenge	0.036		2.189	*P* < 0.05
**Total effect**				
obligation→LS	0.222		3.421	*P* < 0.01
Challenge→LS	0.288		3.988	*P* < 0.001
Control→LS	0.110		2.138	*P* < 0.05
Obligation→PWB	0.200		3.119	*P* < 0.01
Challenge→PWB	0.209		2.739	*P* < 0.01
Control→PWB	0.453		10.343	*P* < 0.001

Ls: life Satisfaction; Pwb: Psychological Well-Being

Table 3 shows the direct, indirect and total effects for the relationship between the family resilience model variables and psychological well-being, life satisfaction and individual resilience. Based on the results of this table, obligation (β = 0.18 and t = 2.89), challenge β = 0.24 and t = 3.11), control (β = 0.05 and t = 1.10), have a significant direct effect on the variance of resilience of pregnant women. Also, obligation (β = 0.17 and t = 2.65), challenge (β = 0.21 and t = 2.90), control (β = 0.11 and t = 2.13) have a significant direct effect on the variance of women’s life satisfaction. Also, obligation (β = 0.17 and t = 2.85), challenge (β = 0.17 and t = 2.82), control (β = 0.45 and t = 10.35), have a significant direct effect on the variance of psychological well-being of pregnant women. Also, the impact of resilience on life satisfaction (β = 0.28 and t = 5.89) and resilience on psychological well-being (β = 0.15 and t = 3.09) were obtained. These results confirm hypotheses 1 to 8 of the study. Therefore, the direct path of obligation, challenge and control on life satisfaction and psychological well-being and the direct path of obligation and challenge on meaningful resilience have been reported. But the control path on resilience was insignificant. Also, individual resilience was significant on life satisfaction and psychological well-being. To test mediation hypotheses, considering the importance of obligation and challenge with individual resilience, as well as their importance and individual resilience with life satisfaction, it can be concluded that individual resilience plays a mediating role between obligation and challenge with life satisfaction. The significance of the indirect effect of obligation and challenge on life satisfaction supported the mediation of resilience, but since control did not show a significant relationship with the mediating variable, it lacked an indirect effect. To test mediation hypotheses, considering the importance of obligation and challenge with individual resilience, as well as their importance and individual resilience with psychological well-being, it can be concluded that individual resilience plays a mediating role between obligation and challenge. Psychological well-being and the significance of the indirect effect of obligation and challenge on psychological well-being through mediation. Individual resilience is supported. But since the control component did not show a significant relationship with the mediating variable, it did not have an indirect effect.

**Fig. 2:**
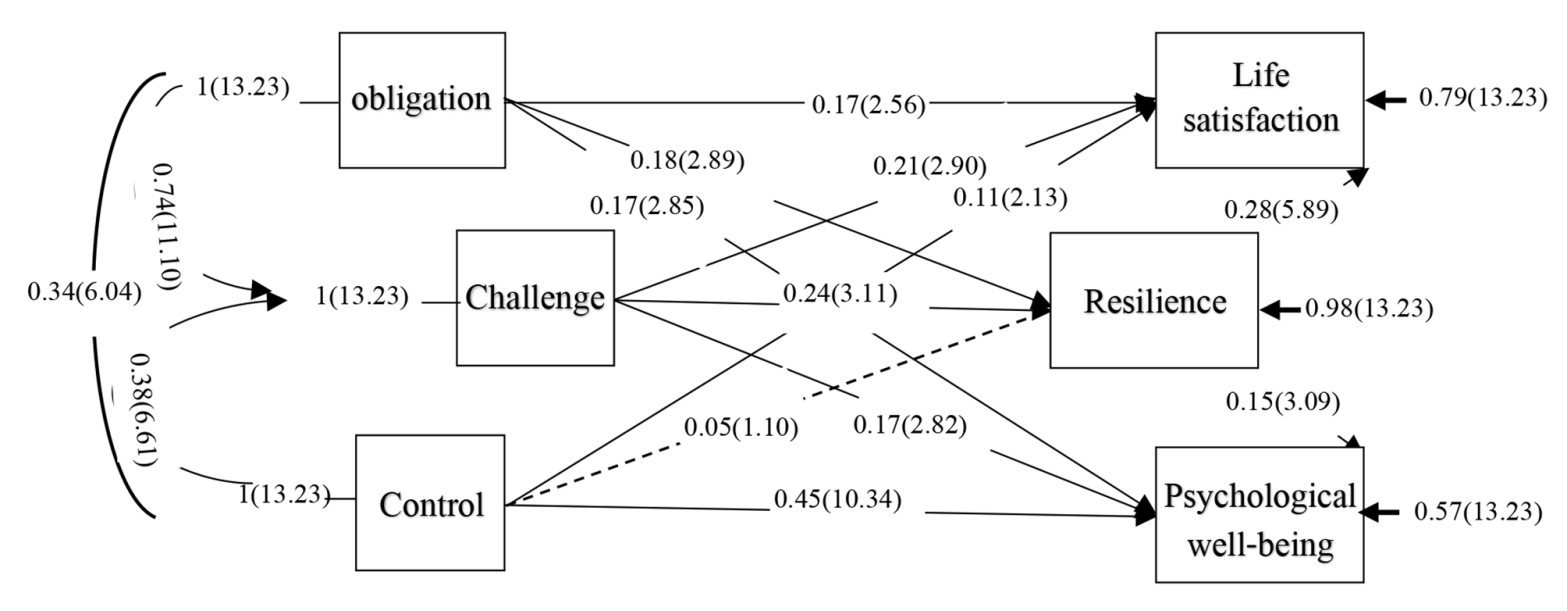
Standard Parameter Values ​​and T Values ​​for Relationships between Variables

Figure 2 shows the relationships obtained from path analysis with the standard parameter index and the t-value (in parentheses) on the paths.

### Model fit indices

Table 4 shows the goodness-of-fit indices of the final path model. The obtained value, acceptance criteria and evaluation result are specified in this table. As can be seen from the table all indicators support the appropriate fit of the model.

**Table 4:** Model Fit Indices

Index	Value	Criteria	Result
CMIN	4.22	-	-
Df	2	-	-
P	0.326	> 0.05	Acceptable
χ2 /df	1.41	< 3	Acceptable
GFI	0.99	> 0.90	Acceptable
AGFI	0.97	> 0.90	Acceptable
TLI	0.97	> 0.90	Acceptable
NFI	0.99	> 0.90	Acceptable
CFI	1	> 0.90	Acceptable
SRMR	0.044	< 0.05	Acceptable
RMSEA	0.019	< 0.05	Acceptable
90%CI	0.028 − 0.010	Close to RMSEA	Acceptable
PCLOSE	0.58	> 0.05	Acceptable

CMIN, chi-square; df, degrees of freedom; GFI, goodness-of-fit index; AGFI, adjusted goodness-of-fit index; TLI: Tucker-Lewis index; NFI, Bentler-Bonett Normed Fit Index; CFI, comparative fit index; SRMR: standardized root mean square residual; RMSEA, root mean square error of approximation; CI, confidence interval; PCLOSE, P value for testing the null hypothesis that the population RMSEA is no greater than 0.05

## Discussion

The purpose of this research was to determine the relationship between family resilience, psychological well-being and life satisfaction with the indirect effect of individual resilience. According to the results of Table 3, the results of path analysis showed that all paths were significant except the path of control to resilience. The data supports the hypothesized relationships between the proposed variables and also the results showed that family resilience is related to individual resilience. This result is consistent with the research of Finkelstein, Pagorek-Eshel and Laufer [15] and Dong Zhu [25]. To explain this finding, it can be said that resilient people generally have more control over life’s adversities and have better problem-solving skills than others, which makes them respond to life’s challenges and crises in a better way and instead of running away from problems. They look at it as a golden opportunity for growth and development and consider changes in life as a natural thing and believe that it is these changes that lead to the improvement and progress of the individual and family members. The another results of this study showed that family resilience has a positive effect on psychological well-being, and this finding is consistent with the studies of Shin and Park [24] and Aivalioti and Pazirkianidis. The coping methods used by the family predict the resilience of the family and the well-being of the family members. To explain this finding, it can be mentioned that the resilience process of the family makes life’s adversities less effective and bearable, and thus it is related to the category of mental health and psychological well-being [30]. Also, during the investigations, it was found that family resilience has a positive effect on life satisfaction in pregnant women, and this [30]finding is in line with the researches of Seo and Hyun [40] and INAL and AKTURK [11]. There is a significant relationship between family resilience and life satisfaction, and in families that have children with special needs, the relationship between these two components is more stable than in families that have children with normal development [11]. In explaining this finding, it can be said that family resilience is a management process that causes the balance of the family not to be disrupted due to the creation of new conditions, and maintaining this balance brings joy and peace among the family members. Resilience is important in a series of people, especially pregnant women; Because these women have become very sensitive and irritable due to hormonal and psychological changes, and with the smallest problem, their peace and balance undergo fundamental changes, which affect the health of the mother and the fetus. The next result shows the existence of a mediating relationship between individual resilience and psychological well-being, which is in line with the researches of Rios-Risquez et al [41], Kim [42] and Labrague [18]. To explain this finding, it can be said that psychological well-being is a characteristic that, as its level increases, it guarantees people’s mental health. This component in life causes people to consider life as meaningful and the meaning and concept of life is valuable for them and to continuously look for opportunities for their growth and development so that they can improve their knowledge and their knowledge and develop their personality. According to the obtained results, the relationship between individual resilience and life satisfaction was confirmed, and this result is consistent with the researches of Limonero [21], Bajaj and Pande [22], Arslan [10], Kim [42] and Labrague [18]. The results of Arslan’s analysis [10] showed that there is a positive and significant relationship between resilience, life satisfaction and self-esteem. To explain this data, it can be mentioned that individual resilience gives a person the strength and ability to overcome crises and challenges and helps the person to show flexible behavior towards that issue so that they can more easily cope with new problems and conditions. Resilience is an ability that we can develop, which makes us improve a series of other abilities at the same time. It can be said that people with higher resilience have meaning and purpose for their lives and never live in vain.

## Conclusion

The data of the present study confirmed the relationship between family resilience, psychological well-being and life satisfaction and the indirect effect of individual resilience. According to these conditions in the life of pregnant women, it can be said that improving resilience can play a significant role in bearing the problems and problems created during this period, which by reducing the pressure caused by the prevailing psychological problems and conditions, preserves them and keeps them healthy. improve their mental health and be more satisfied with life, spend this period more peacefully and ensure their health and the health of the fetus in the future. One of the applications of the research results is for couples whose spouses are pregnant and also for all health professionals who are engaged in the field of mental health counseling, it can be of great help in controlling mental pressure and providing training to pregnant women Through educational courses for pregnant mothers and mothers who intend to become pregnant, encourage them to study in fields related to their conditions in order to avoid serious damage to their psyche.

## Research limitations

This research was conducted in the city of Zanjan, Iran, and the subject of this research was pregnant women. Due to the cultural differences in different cities, one should be cautious in generalizing the results to other cities and other cultures. In this study, the tool used was a questionnaire, which can be said that the use of a questionnaire has limitations and the respondents may not state the facts about themselves for various reasons, and another limitation of this research is the lack of control of environmental factors. As in some cases, such as in the hospital, several pregnant women completed the questionnaires together, it is possible that she did not complete the questionnaire under the influence of another woman and according to the existing facts. Another limitation of this research was that it was conducted during the Covid-19 pandemic and may have affected the results of the research.

## Data Availability

The datasets during and/or analyzed during the current study available from the corresponding author on reasonable request.
